# Impact of Ejection Fraction on long-term outcome after ST-Elevation Myocardial Infarction - comparison between cardiac magnetic resonance imaging and transthoracic echocardiography

**DOI:** 10.1186/1532-429X-14-S1-O15

**Published:** 2012-02-01

**Authors:** Suzanne de Waha, Steffen Desch, Ingo Eitel, Philipp Lurz, Georg Fuernau, Matthias Gutberlet, Gerhard Schuler, Holger Thiele

**Affiliations:** 1 Department of Internal Medicine/Cardiology, Heart Center Leipzig, Leipzig, Germany; 2 Department of Diagnostic and Interventional Radiology, Heart Center Leipzig, Leipzig, Germany

## Summary

EF assessed by echocardiography and EF assessed by CMR are only moderately correlated. The exact quantification of EF by CMR provides additional prognostic information and may affect further treatment strategies, such as intensification of medication including aldosterone antagonist use or even device therapy.

## Background

Left ventricular ejection fraction (EF) is a strong prognostic marker for clinical outcome after ST-elevation myocardial infarction (STEMI). In clinical practice echocardiography is most commonly used to assess EF, although cardiac magnetic resonance imaging (CMR) might have higher accuracy. However, it remains unclear if measuring EF by CMR in comparison to echocardiography provides incremental prognostic information for the assessment of long-term outcome in patients with STEMI.

## Methods

STEMI patients reperfused by primary angioplasty (n=392) within 12 hours after symptom onset underwent transthoracic echocardiography and CMR 3 days after the index event (interquartile range [IQR] 2-4). Assessment of EF by echocardiography (EFecho) was performed in standard apical 4-chamber view. Standard cine steady-state-free-precession sequences in short-axis views were carried out to measure EF by CMR (EFcmr). The corresponding EF was graded as severely decreased (<35%), mildly to moderately decreased (35-55%) and normal (>55). Clinical follow-up was conducted after 19 months (IQR 10-27). The primary endpoint was defined as a composite of death and congestive heart failure.

## Results

EFecho was 45% (IQR 40-55), whereas EFcmr was 50% (IQR 40-58). EFecho was only moderately correlated to EFcmr (r=0.51, p<0.001). Both EFecho and EFcmr were significantly associated with the occurrence of the primary endpoint (EFecho: hazard ratio [HR] 0.96, 95%CI 0.94-0.98, p<0.001 / EFcmr: HR 0.94, 95%CI 0.93-0.95, p<0.001). Comparison of a risk prediction model including only EFecho to a model including EFcmr on top of EFecho resulted in a significant increase of the c-statistics from 0.65 to 0.69 (p=0.04) demonstrating therefore an incremental prognostic value of EFcmr. Finally, EFcmr led to re-grading of 144 patients (36.7%): 122 patients were re-classified to a group with less severely impaired EF, whereas only 22 patients displayed a higher grade of decrease in EF.

## Conclusions

EFecho and EFcmr are only moderately correlated. The exact quantification of EF by CMR provides additional prognostic information and may affect further treatment strategies, such as intensification of medication including aldosterone antagonist use or even device therapy.

## Funding

None.

